# Prognóstico após Infarto do Miocárdio – Um Olhar Profundo sobre o Tecido Miocárdico

**DOI:** 10.36660/abc.20220798

**Published:** 2022-11-23

**Affiliations:** 1 Departamento de Cardiologia Hospital de Santa Marta Lisboa Portugal Departamento de Cardiologia, Hospital de Santa Marta, Lisboa – Portugal

**Keywords:** Infarto Agudo do Miocárdio, Remodelação Ventricular, Ressonância Magnética, Metaloproteinases

A remodelação cardíaca adversa após infarto agudo do miocárdio (IAM), independentemente da intervenção coronária percutânea primária, está fortemente associada ao desenvolvimento de insuficiência cardíaca e mau prognóstico. Como as características demográficas e clínicas não são suficientemente sensíveis para predizer remodelação adversa após IAM, são necessários parâmetros mais precisos para identificar indivíduos em risco de progressão para disfunção ventricular e insuficiência cardíaca, potencialmente permitindo uma terapia precoce e intensiva modificadora do prognóstico.

Surgiram novos biomarcadores de remodelação cardíaca adversa, como as metaloproteinases de matriz (MMP) 2 MMP-2, MMP-6, MMP-9.^1^ Há uma crescente conscientização da importância do interstício na fisiopatologia das doenças cardíacas além dos miócitos cardíacos. De fato, o interstício cardíaco representa um terço do volume total do miocárdio. Contém dois terços do número total de células do miocárdio, principalmente fibroblastos.^2^ Os fibroblastos são responsáveis pela manutenção da homeostase intersticial, e a produção de MMP.^3^ A expansão intersticial está associada a efeitos adversos na função miocárdica em múltiplas entidades.^4–6^

A RMC é hoje um importante método de avaliação do miocárdio. O mapeamento paramétrico é uma técnica de RMC que quantifica diretamente o tempo de relaxamento T1 de cada voxel dentro de uma imagem de RMC, construindo um mapa visual e permitindo uma avaliação não invasiva do tecido miocárdico.^7^ A correlação entre os valores de T1 e o volume extracelular (VEC) com fração de volume de colágeno foi validada com biópsia miocárdica.^8^

Ferhat Eyyupkoca et al.^9^ mostraram que existe uma diferença significativa nas características dos tecidos entre pacientes com e sem remodelação adversa desde o período inicial após a fase aguda do IAM. Curiosamente, na RMC inicial, os volumes ventriculares esquerdos e a função sistólica foram semelhantes entre os pacientes com e sem remodelação adversa, ressaltando a importância de marcadores adicionais, como o estudo do espaço extracelular, para predizer desfechos. O VEC e o volume da matriz (VMi) avaliados pela RMC foram significativamente diferentes no exame feito em duas semanas, e a magnitude da diferença foi ainda maior após 6 meses refletindo a remodelação adversa. Embora o VEC tenha aumentado no acompanhamento em ambos os grupos, o índice de volume da matriz aumentou apenas no grupo de remodelação adversa, destacando seu valor agregado no estudo abrangente do espaço extracelular comparado ao VEC. Além disso, o modelo com o ΔMVi teve um desempenho melhor do que o modelo ΔECV, mostrando melhor sensibilidade e especificidade para prever remodelação adversa.Por outro lado, o índice de volume celular diminuiu em ambos os grupos de estudo sem diferença entre eles. Um ponto forte deste trabalho é a evidência da boa correlação entre MMP-2 e MVi, e VEC, o que contribui para fortalecer a associação entre RMC e biomarcadores de fibrose e alterações teciduais miocárdicas.

Na era do aumento dos biomarcadores e parâmetros de imagem disponíveis, é fundamental usar cuidadosamente todas as informações para aplicar a abordagem mais eficiente para prever o prognóstico na prática clínica. Considerando a elevada incidência de IAM e o ônus do desenvolvimento de insuficiência cardíaca para pacientes e sistemas de saúde, um modelo que antecipe a remodelação mal adaptativa pode influenciar a remodelação miocárdica com a implementação precoce de intervenções farmacológicas. Com a progressiva disseminação da RMC e da técnica de mapeamento, isso pode em breve assumir o papel de triagem para remodelação adversa após IAM. Nesta edição do ABC, Ferhat Eyyupkoca et al. contribuem a aumentar a precisão da avaliação de pacientes com infarto do miocárdio, adicionando MVi à análise de RMC, que é fácil de obter a partir de sequências de mapeamento usadas rotineiramente e pode ser facilmente adotada em unidades de RMC. Este parâmetro promissor precisa de validação adicional em amostras maiores e populações mais heterogêneas.
