# Supplementary material for: Prognóstico após Infarto do Miocárdio – Um Olhar Profundo sobre o Tecido Miocárdico
Source: Arq Bras Cardiol. 2022 Nov 23;119(6):958–9. [Article in Portuguese] doi: 10.36660/abc.20220798 (PMC9814801; doi:10.36660/abc.20220798)
Supplement: Translation

# Translation: Prognosis after Myocardial Infarction – A Deep Look into Myocardial Tissue

**Affiliations:** 1 Lisboa Portugal Departamento de Cardiologia, Hospital de Santa Marta, Lisboa – Portugal

**Keywords:** Infarto Agudo do Miocárdio, Remodelação Ventricular, Ressonância Magnética, Metaloproteinases

Adverse cardiac remodeling after acute myocardial infarction (MI), regardless of primary percutaneous coronary intervention, is strongly associated with the development of heart failure and poor prognosis. Since demographic and clinical characteristics are not sufficiently sensitive to predict adverse remodeling after MI, more precise parameters are needed to identify individuals at risk of progression to ventricular dysfunction and heart failure, potentially allowing an early and intensive prognosis modifying therapy.

New biomarkers of adverse cardiac remodeling have emerged, such as matrix metalloproteinases (MMP) 2 MMP-2, MMP-6, MMP-9.^1^ There is an increasing awareness of the importance of interstitium in the pathophysiology of cardiac diseases beyond cardiac myocytes. In fact, the cardiac interstitium represents one-third of the total myocardial volume. It contains two-thirds of the total number of cells in the myocardium, mainly fibroblasts.^2^ Fibroblasts are responsible for maintaining interstitial homeostasis, and the production of MMP.^3^ Interstitial expansion is associated with adverse effects on myocardial function in multiple entities.^4–6^

CMR is nowadays an important method to evaluate the myocardium. Parametric mapping is a CMR technique that directly quantifies the T1 relaxation time of each voxel within a CMR image, building a visual map and allowing a non-invasive evaluation of myocardial tissue.^7^ The correlation between T1 values and extracellular volume (ECV) with collagen volume fraction has been validated with myocardial biopsy.^8^

Ferhat Eyyupkoca et al.^9^ showed that there is a significant difference in tissue characteristics between patients with and without adverse remodeling from the early period after the acute phase of MI. Interestingly, in the early CMR, left ventricular volumes and systolic function were similar between patients with and without adverse remodeling, emphasizing the importance of additional markers, as the study of extracellular space, for predicting outcomes. ECV and matrix volume (MVi) assessed by CMR were significantly different in the exam done at two weeks, and the magnitude of the difference was even higher after 6 months reflecting the adverse remodeling. Although ECV increased in the follow-up in both groups, the matrix volume index just increased in the adverse remodeling group, highlighting its added value in the comprehensive study of extracellular space compared to ECV. Furthermore, the model with ΔMVi performed better than the ΔECV model, showing better sensibility and specificity to predict adverse remodeling. On the other hand, the cell volume index decreased in both study groups without difference among them. A strong point of this work is the evidence of the good correlation between MMP-2 and MVi, and ECV, which contributes to strengthening the association between CMR and biomarkers of fibrosis and myocardial tissue changes.

In the era of increasing available biomarkers and imaging parameters, it is paramount to thoughtfully use all the information to apply the most efficient approach to predict prognosis in clinical practice. Considering the elevated incidence of MI and the onus of the development of heart failure for patients and health systems, a model that anticipates maladaptive remodeling can influence myocardial remodeling with the early implementation of pharmacological interventions. With the progressive widespread of CMR and mapping technique, this may soon assume the screening role for adverse remodeling after MI. In this issue of the Journal, Ferhat Eyyupkoca et al. contribute to increasing the accuracy of the evaluation of MI patients by adding MVi to CMR analysis, which is easy to obtain from routinely used mapping sequences and may be easily adopted at CMR units. This promising parameter needs further validation in bigger samples and more heterogeneous populations.
